# Luminance-polarity distribution across the symmetry axis affects the electrophysiological response to symmetry

**DOI:** 10.1016/j.neuroimage.2018.02.008

**Published:** 2018-06

**Authors:** Damien Wright, Claire Mitchell, Benjamin R. Dering, Elena Gheorghiu

**Affiliations:** University of Stirling, Department of Psychology, Stirling, FK9 4LA, Scotland, United Kingdom

**Keywords:** Luminance-polarity, Colour, Symmetry, EEG, ERP, Sustained Posterior Negativity, Microstates

## Abstract

Electrophysiological studies of symmetry have found a difference wave termed the Sustained Posterior Negativity (SPN) related to the presence of symmetry. Yet the extent to which the SPN is modulated by luminance-polarity and colour content is unknown. Here we examine how luminance-polarity distribution across the symmetry axis, grouping by luminance polarity, and the number of colours in the stimuli, modulate the SPN. Stimuli were dot patterns arranged either symmetrically or quasi-randomly. There were several arrangements: ’*segregated*’-symmetric dots were of one polarity and randomly-positioned dots were of the other; ‘*unsegregated*’-symmetric dots were of both polarities in equal proportions; ‘*anti-symmetric*’-dots were of opposite polarity across the symmetry axis; ‘*polarity-grouped anti-symmetric*’-this is the same as anti-symmetric but with half the pattern of one polarity and the other half of opposite polarity; *multi-colour symmetric* patterns made of two, three to four colours. We found that the SPN is: (i) reduced by the amount of position-symmetry, (ii) sensitive to luminance-polarity mismatch across the symmetry axis, and (iii) not modulated by the number of colours in the stimuli. Our results show that the sustained nature of the SPN coincides with the late onset of a topographic microstate sensitive to symmetry. These findings emphasise the importance of not only position symmetry, but also luminance polarity matching across the symmetry axis.

## Highlights

• SPN is reduced by luminance-polarity mismatch across the symmetry axis for 50% position symmetry.
• SPN is modulated by the amount of positional symmetry in the stimulus.
• SPN is not modulated by the number of colours in the stimuli.
• SPN may be driven by the late onset of symmetry sensitive mechanisms.
• Symmetry detection in anti-symmetric patterns can benefit from grouping by luminance polarity.

## Introduction

**Fig. 1:**
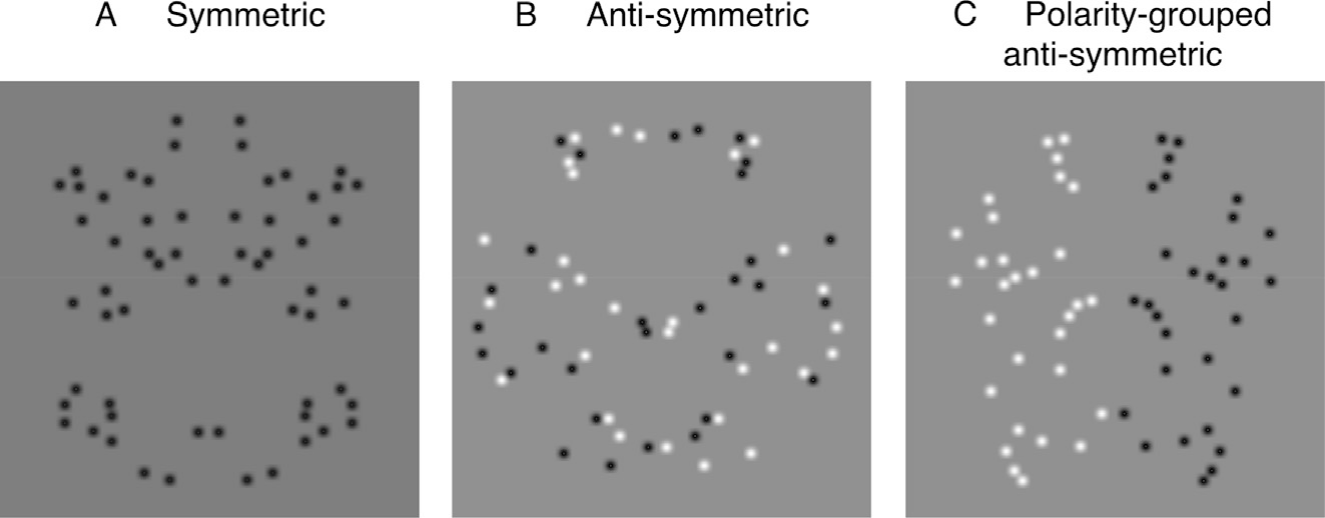
**Examples of 100% position-symmetric patterns with different luminance-polarity arrangements**. (A) Symmetric pattern in which elements across the vertical axis are matched in luminance polarity. (B) Anti-symmetric pattern in which the matched pairs across the symmetry axis have opposite luminance-polarity. (C) Polarity-grouped anti-symmetric pattern in which each half of the pattern is of different luminance-polarity. Note, each of the patterns contains the same amount of positional information but they differ in regard to their luminance-polarity arrangement.

Symmetry, a ubiquitous feature in natural scenes, is found in both biological and man-made objects, and is detected effortlessly by the human visual system. Mirror-symmetry (henceforth just ‘symmetry’) occurs when half of a pattern reflects the other about a vertical axis. Psychophysical and brain imaging studies have shown that symmetry plays a central role in object recognition (Vetter et al., 1994) and figure-ground segmentation (i.e. symmetric regions tend to be seen as figure rather than ground) (Driver et al., 1992) and, it involves a large network of extrastriate visual areas such as V3a, V4, V7 and LOC (Cattaneo et al., 2011, Chen et al., 2007, Sasaki et al., 2005). Event-Related Potential (ERP) studies of symmetry have revealed that amplitude in posterior electrodes is lower for symmetric than quasi-random patterns from ∼200 ms after stimulus onset, thus resulting in a difference wave termed the Sustained Posterior Negativity (SPN) that reportedly indexes symmetry perception (Bertamini and Makin, 2014, Jacobsen and Höfel, 2003, Norcia et al., 2002). However, most brain imaging and ERP studies have employed perfectly symmetric stimuli in which symmetry is defined in terms of both position and luminance polarity of elements (see Fig. 1A). This contrasts with most objects in natural scenes, which despite their positional or spatial form symmetry, often vary in the strength of symmetry due to differences in visual features such as colour and luminance polarity. While recent psychophysical (behavioural) studies have examined the role of luminance-polarity and colour (Gheorghiu et al., 2016, Morales and Pashler, 1999, Wu and Chen, 2014, Wu and Chen, 2017) in symmetry perception, very little is known about how these visual attributes influence the electrophysiological correlates of symmetry perception. In this communication, we use ERP methods to investigate how luminance-polarity distribution across the symmetry axis, grouping by luminance-polarity, and the number of colours in the stimuli affect the specific SPN response to symmetry.

Although psychophysical (behavioural) studies have investigated the effects of luminance polarity on symmetry perception by comparing performance in dot (or Gaussian blob) patterns in which position-symmetric elements have either the same or different luminance-polarity across the symmetry axis (Brooks and van der Zwan, 2002, Gheorghiu et al., 2016, Mancini et al., 2005, Zhang and Gerbino, 1992), the extent to which this affects the ERP response to symmetry remains unclear. Most behavioural studies found that performance was better when symmetrical dot-pairs had the same luminance-polarity (either black or white) or the whole pattern was of one polarity and dropped to near chance level with anti-symmetric patterns (i.e. position-symmetric elements mismatched in luminance polarity across the symmetry axis – see Fig. 1B) thus, demonstrating that symmetry mechanisms are sensitive to luminance polarity (Brooks and van der Zwan, 2002, Zhang and Gerbino, 1992). Performance was also poor with anti-symmetric patterns in which *all* dots were of one luminance-polarity on one side of the symmetry axis and opposite polarity on the other side (i.e. symmetric halves were of opposite luminance-polarity – Fig. 1C) suggesting that grouping by luminance polarity does not improve performance (Zhang and Gerbino, 1992).

Yet, the sensitivity to luminance-polarity has not been consistently found across all studies. A few studies used low dot-density patterns and reported similar performance levels when the symmetrical dot-pairs have the same or different luminance-polarity across the symmetry axis (Mancini et al., 2005, Tyler and Hardage, 1996, Wenderoth, 1996), thus, suggesting no role of luminance-polarity in symmetry detection. However, Mancini et al. (2005) argue that the equal sensitivity to symmetry and anti-symmetry found in these studies might not be due to the fact that they elicit similar responses from spatial-filtering models (Rainville and Kingdom, 1999, Rainville and Kingdom, 2000, Dakin and Hess, 1997, Dakin and Watt, 1994) involving second-order channels. Instead, they posit that sensitivity to symmetry arises from filtering models involving quasi-linear channels whereas sensitivity to anti-symmetry arises from *attentional mechanisms* that operate in low dot-density displays by registering the *positional symmetry* of individual dots that differ in luminance-polarity. However, this type of attentional resource is different from *feature-based* attention (i.e. attention to colour/luminance polarity), which has been found to contribute to symmetry perception (Gheorghiu et al., 2016). Recently, Gheorghiu et al. (2016) have shown that symmetry detection improves when the observers knew beforehand the luminance-polarity or colour of the symmetric pattern thus, demonstrating that symmetry detection mechanisms can benefit from feature-based (i.e. luminance-polarity/colour) attention.

With regards to the effect of luminance-polarity on the electrophysiological response to symmetry, only one single study, Makin et al. (2016), has examined the ERP responses to symmetry by comparing anti-symmetric with symmetric dot-patterns in which matched-pairs were of both luminance polarities. Makin et al. (2016) found similar SPN responses with symmetric and anti-symmetric patterns for one-fold symmetry (i.e. vertical axis of symmetry) and slightly reduced SPNs with anti-symmetric patterns having four-fold symmetry. It is important to note that in Makin et al. (2016) study, stimuli were of variable dot-density and unbalanced in terms of luminance polarity (i.e. contained unequal number of white and black dots) from *trial to trial* (see their Fig.13) although, *on average* across all trials and stimulus conditions, they were of roughly equivalent dot density (mean number of dots 172 ± 15), and luminance polarity ratio. However, it is likely that the *trial by trial* variability in dot density and luminance polarity might have affected Makin et al. results given that dot density affects symmetry detection in *anti-symmetric* patterns (Mancini et al., 2005, Tyler and Hardage, 1996, Wenderoth, 1996) and unbalanced number of dots in the two polarities might result in a 'pop-out' effect and hence, selective attention to one luminance polarity over another (Morales and Pashler, 1999, Gheorghiu et al., 2016). Makin et al. (2016) concluded that symmetry perception feeds on both first (luminance-sensitive) and second order (contrast-sensitive) channels with the SPN indexing a post-filter neural response to symmetry. Thus, it remains unclear how luminance-polarity distribution across the symmetry axis affects the SPN response to symmetry. In this study, we will examine this issue in detail.

With regards to colour, a number of studies have found that symmetry detection mechanisms, while sensitive to colour-correlations across the symmetry axis and subject to the benefits of attention-to-colour, are not colour selective (Gheorghiu et al., 2016, Morales and Pashler, 1999). Symmetry detection was also found to be affected by the number of colours in the pattern (Morales and Pashler, 1999, Wu and Chen, 2017).Using non-isoluminant patterns consisting of 16 coloured squares arranged either symmetrically or with some mismatched in colour across the vertical axis, Morales and Pashler (1999) compared reaction times for detecting symmetry in two and four colour patterns. They found that reaction times were slower and less accurate for four-colours than to two-colours patterns, suggesting that symmetry could only be detected by switching attention from one colour to the next. Conversely, Wu and Chen (2017) reported that symmetry detection is facilitated by increasing the number of colours in the stimuli (i.e. symmetry detection thresholds decreased as the number of colours increased). Thus, it remains unclear how the number of colours affects symmetry perception. Further, there are no electrophysiological studies that have examined how the number of colours in the symmetric pattern affects the SPN response to symmetry.

In this communication, we examine how luminance-polarity distribution across the symmetry axis, grouping by luminance polarity in anti-symmetric patterns and number of colours in the stimuli modulate the electrophysiological response to symmetry. In conjunction with ERPs, we examine functional topographic microstates, that is, the quasi-stable periods of synchronised neural activity which allow one to gain insight into topographic changes across time (Lehmann et al., 1998). These topographic changes allow us to infer if the brain's response to symmetry, i.e. the SPN, employs fundamentally different neural source generators when viewing symmetry compared to noise conditions, which is currently unknown.

**Fig. 2:**
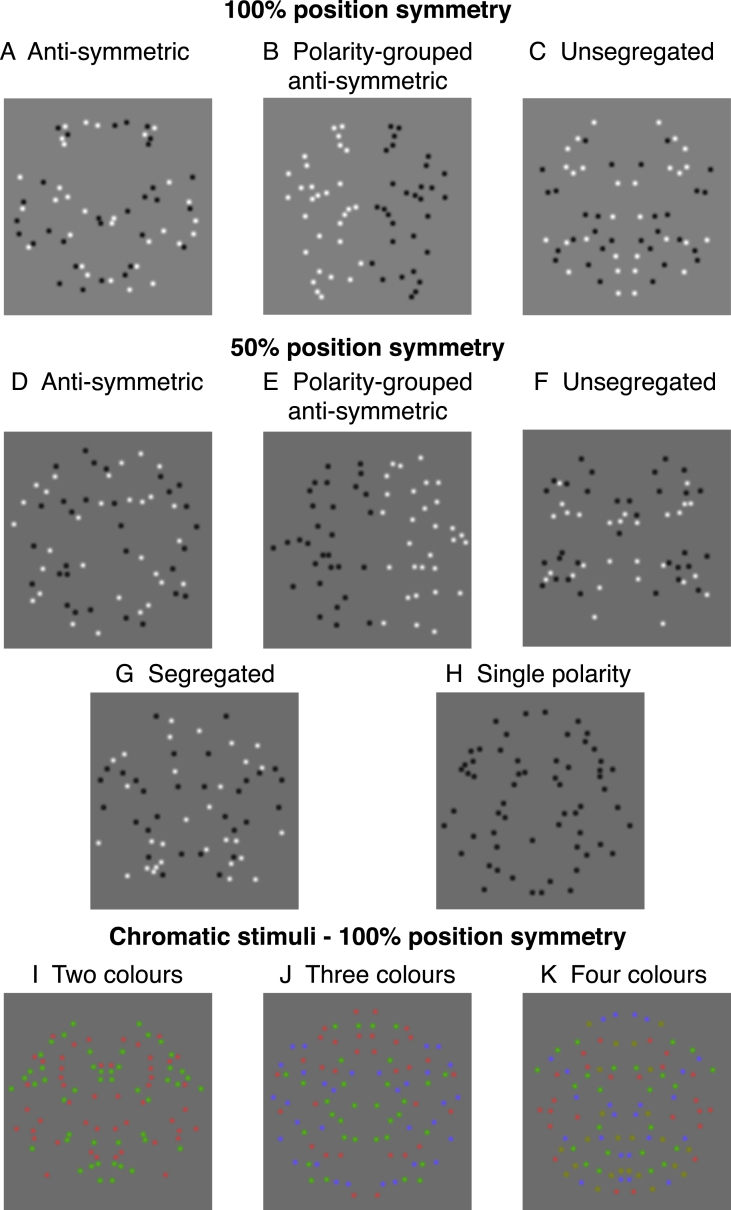
**Examples of 100% position-symmetric stimuli used in Experiment 1 (A**–**C), 50% position-symmetric stimuli used in Experiment 2 (D**–**H), and chromatic stimuli used in Experiment 3 (I**–**K).** A) Anti-symmetric patterns in which position-symmetric dots were of opposite luminance-polarity across the symmetry axis; B) Polarity-grouped – the same as the anti-symmetric condition but with one half of the pattern of one luminance polarity and the other of opposite polarity; C) Unsegregated – symmetric-pairs were the same luminance polarity, with an equal number of black and white dot pairs. (D–H) Examples of 50% position-symmetric patterns used in Experiment 2: (D) Anti-symmetric; (E) Polarity-grouped anti-symmetric; (F) Unsegregated; (G) Segregated in which symmetrical pairs are black and noise dots are white; (H) Single polarity patterns. In this experiment, we also used single polarity patterns with 100% position symmetry (not shown). (I–K) Examples of 100% position-symmetric chromatic stimuli made of two (I), three (J) or four (K) colours.

**Fig. 3:**
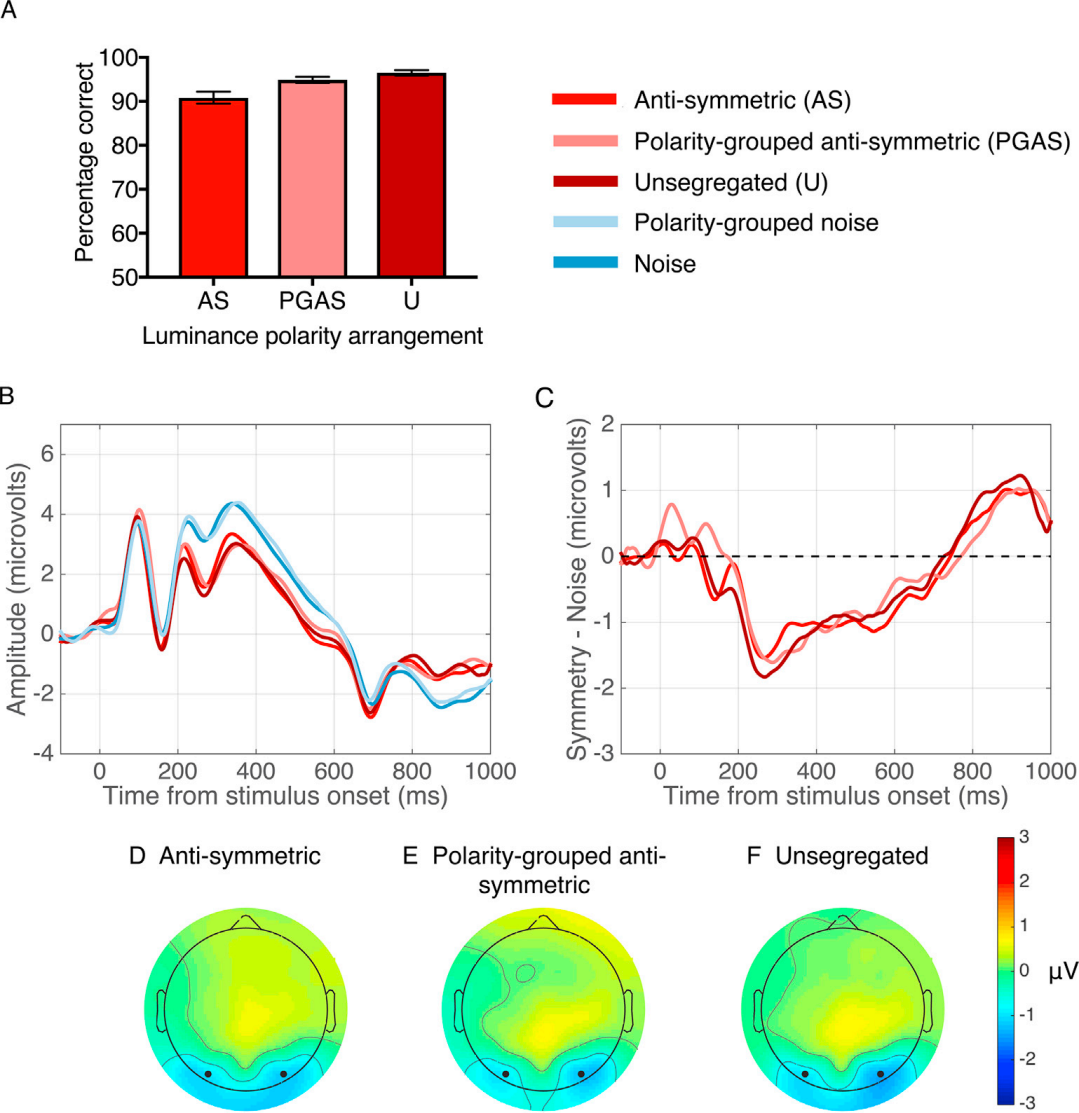
**Results for Experiment 1.** (A) Performance (% correct answers) in the symmetry detection task with symmetric, anti-symmetric and luminance-polarity grouped patterns containing 100% position symmetry. (B) Grand-average ERPs for anti-symmetric (red), polarity-grouped anti-symmetric (pink), unsegregated (burgandy), noise (blue) and polarity-grouped noise (light blue) conditions. (C) Difference waves (Symmetry – Noise) for each condition. Waveforms depict the average of electrodes PO7 and PO8. (D–F) Topographic difference map for anti-symmetric condition (D), polarity-grouped anti-symmetric (E) and unsegregated (F). Each topographic difference map shows the difference between symmetry and noise in the 200–600 ms time window. Black dots indicate the position of electrodes PO7 and PO8.

In Experiment 1, we use 100% position-symmetric patterns under three luminance-polarity arrangements: (1) *anti-symmetric* – in which position-symmetric dots were of opposite luminance-polarity across the symmetry axis (Fig. 2A); (2) *polarity-grouped anti-symmetric* – these were anti-symmetric patterns in which all elements were of one luminance-polarity (i.e. white) on one side of the symmetry axis and opposite (i.e. black) luminance-polarity on the other (Fig. 2B); (3) *unsegregated* – symmetric pairs were of the same luminance-polarity (either white or black) in equal proportions (Fig. 2C). Perfect symmetric patterns (100% position-symmetry signal, 0% noise) do not allow one to examine whether symmetry mechanisms are gated by (or selective to) luminance polarity since the symmetry signal is in both luminance polarities and thus, the brain's response is optimal irrespective of luminance polarity. Therefore, in Experiment 2, we used patterns in which the symmetry and noise dots were *segregated* by luminance polarity (i.e. symmetrical dots were of one luminance polarity and noise dots of the other – Fig. 2G), and this is possible only when the patterns contain 50% position symmetry. In this experiment, there were five luminance-polarity arrangements: anti-symmetric, polarity-grouped anti-symmetric, unsegregated, segregated (i.e. the symmetry signal was of one luminance polarity whilst the noise was the other) and single polarity (i.e. all dots were of the same luminance polarity with the symmetric patterns having either 50% or 100% position symmetry). This experiment will allow us to compare directly between various luminance-polarity arrangements and reveal how luminance polarity modulates the ERPs response to symmetry. For both Experiment 1 and 2, we predict that if the ERP response to symmetry is *sensitive* to luminance-polarity across the symmetry axis (i.e. across-the-symmetry-midline *positional correlations* are affected by the mismatch in luminance polarity), then we expect to find a difference in the amplitude of the SPN response to anti-symmetric and unsegregated patterns. In addition, if grouping by luminance-polarity modulates the ERP response to anti-symmetry, then we expect to find differences in ERP responses to anti-symmetric and polarity-grouped anti-symmetric patterns.

It is also worthwhile to note the difference between *sensitivity* and *selectivity* to luminance polarity of symmetry mechanisms. Luminance-polarity*-selective* symmetry mechanisms imply the existence of *positional-grouping* mechanisms that are *gated* by luminance polarity, that is, a symmetry mechanism that groups all white elements together to derive a ‘white-symmetry’ signal and another mechanism that groups all black elements in the pattern to derive a ‘black-symmetry’ signal. In previous studies, Gheorghiu et al. (2016) demonstrated that symmetry mechanisms pool *all signals* from *luminance-polarity-correlated* elements across the symmetry axis, that is, there is just one channel that pools on and off luminance-polarity signals from luminance-polarity-correlated elements across the symmetry axis. Therefore, considering that symmetry mechanisms are *not selective* to luminance-polarity (Gheorghiu et al., 2016), we predict comparable SPNs between segregated and unsegregated conditions.

In Experiment 3, we will examine the effect of number of colours by using 100% position-symmetric patterns containing two (red, green), three (red, green, blue) or four (red, green, blue, yellow) colours. If symmetry mechanisms are not selective to chromatic information then we predict no differences in SPN amplitude between two, three and four colours.

Finally, if the SPN difference reflects neural mechanisms specific to symmetry perception, we should see across all three experiments topographic microstates that reflect symmetry processing only, which are not present in comparable noise conditions.

## Methods

### Participants

A total of seventy-two participants (24 participants for each experiment) who were naive with regard to the experimental aims took part in this study. All observers had normal or corrected-to-normal visual acuity and normal colour vision. Observers gave their written informed consent prior to participating in the study and were treated in accordance with the Declaration of Helsinki. The research protocol was approved by the University of Stirling Psychology Ethics Committee.

### Stimuli – generation and display

Stimuli were dot patterns presented on a gamma-corrected 19” TFT monitor at 60 Hz frame rate and 1024 × 768 spatial resolution in a dark, sound attenuated room. All stimuli were presented in the centre of the monitor on a mid-grey background with mean luminance of 65.5 cd/m^2^. The stimuli had a diameter of 12 deg and were made of 60 achromatic Gaussian dots with a standard deviation of 0.08 deg and a Gaussian size standard deviation factor of 5.

In Experiment 1, we used patterns made of achromatic Gaussian dots that were positioned either symmetrically (100% position symmetry) or quasi-randomly. There were three symmetrical stimulus conditions: (1) *anti-symmetric,* in which the symmetric dots were of opposite luminance polarity across the symmetry axis – see Fig. 2A; (2) *polarity-grouped anti-symmetric* – this was the same as the anti-symmetric condition but position symmetric dots were of one luminance-polarity on one side of the symmetry axis (i.e. either white or black dots) and opposite luminance-polarity on the other (i.e. either black or white) – see Fig. 2B; (3) *unsegregated,* in which symmetric pairs were of both luminance polarities in equal proportions – see Fig. 2C.

In Experiment 2, we used patterns in which 50% of the Gaussian dots were arranged symmetrically in the symmetric conditions, while the remaining 50% dots were randomly positioned and drawn equally from both luminance polarities. As in Experiment 1, we used (1) *anti-symmetric* (i.e. symmetric dots were of opposite luminance polarity across the symmetry axis and noise dots drawn equally from both luminance polarities – see Fig. 2D), (2) *polarity-grouped* *anti-symmetric* (i.e. the same as anti-symmetric but with all dots of one luminance-polarity on one side of the symmetry axis and the opposite polarity on the other side – see Fig. 2E) and (3) *unsegregated* (i.e. symmetric dots were of both polarities in equal proportions, as were the noise dots – see Fig. 2F). In addition to these conditions, we used a (4) *segregated* condition in which the symmetric dots were of one luminance polarity, but the polarity was randomly selected on each trial between white and black, while the noise dots were of the other polarity – see Fig. 2G, and (5) *single luminance-polarity* symmetrical patterns of 50% (see Fig. 2H) and 100% position symmetry.

For both Experiment 1 and 2, the quasi-randomly pattern contained an equal number of black and white dots as the symmetrical patterns. For Experiment 1, there were two types of noise patterns: (a) randomly distributed black and white dots, and (b) polarity-grouped noise in which half of the random pattern was of one luminance polarity (either white or black) and the other was of opposite polarity (either black or white). In addition to these two types of noise patterns, in Experiment 2 we used single polarity noise in which all dots that were either white or black.

In Experiment 3, we used fully-symmetric, non-isoluminant chromatic patterns made of 96 Gaussian dots. The patterns contained two (red and green), three (red, green and blue) or four (red, green, blue and yellow) colours (Fig. 2I–K). The quasi-randomly pattern contained an equal number of coloured dots as the symmetrical patterns.

### Procedure – 2IFC

In all experiments, a two-interval forced-choice (2IFC) procedure was used to measure symmetry detection. On each trial, a stimulus corresponding to one of the symmetric conditions was randomly presented in one of the two intervals while the other interval contained a noise stimulus made up of quasi-randomly positioned dots (i.e. null interval) of both luminance polarities in equal proportions. For the polarity-grouped anti-symmetric condition, we used a polarity-grouped noise pattern while for the single-polarity condition, we used a single polarity noise pattern. Patterns were presented for 500 ms with an inter-stimulus interval of 500 ms.

Participant's task was to indicate which interval contained the symmetric pattern by responding via a key press. In each of the three experiments (Experiment 1, 2 and 3), each condition was presented 100 times with the stimulus conditions presented in random order. This resulted in a total of 300 trials in Experiment 1, 600 trials in Experiment 2 and 300 trials in Experiment 3. The experiments were divided into blocks of 50 trials to allow the participant regular breaks and for the electrodes to be checked.

### Procedure – EEG recording and analysis

Electroencephalogram (EEG) signals were recorded using a SynAmps 2 amplifier and Scan 4.5 software (Neuroscan Inc., EL Paso TX, USA). Four external channels recorded bipolar horizontal and vertical electro-oculograph (EOG) signals. For all three experiments, raw electroencephalogram (EEG) signals were recorded from the scalp at a 1 kHz sampling rate from 64 Ag/AgCl electrodes positioned according to the extended 10–20 system and using CZ as the online reference. All electrode recording impedances were kept below 5 KΩ. The electroencephalogram was filtered in real time (i.e. on-line) between 0.01 and 200 Hz and off-line with a low pass 40 Hz filter (48 db/octave slope). Eye blink artefacts were mathematically corrected using a model blink artefact computed for each individual based upon the method of Gratton et al. (1983). Signals exceeding ±100 μV in any given epoch were automatically discarded. We rejected on average 6 trials out of 100 per condition, for every participant across all three experiments (Experiment 1: 8.14 ± 12.33% of trials; Experiment 2: 4.66 ± 7.2% of trials; Experiment 3: 5.08 ± 4.92% of trials). EEG recordings were cut into epochs ranging from −100 ms to 1000 ms, with a baseline of −100 to 0 ms, after stimulus onset and averaged for each individual according to the experimental conditions. Grand-averages for each experiment were calculated after re-referencing individual participant ERPs to the common average reference. In each experiment, the SPN was defined as the difference between symmetric and quasi-random patterns from 200 ms to 600 ms after stimulus onset and measured from electrodes PO7 and PO8.

### Procedure - topographic analysis and functional microstates

For each experiment, the EEG data were subjected to further topographical analyses to look for stable patterns of scalp activity, which was performed using Cartool software (Brunet et al., 2011; brainmapping.unige.ch/cartool). The topography of the scalp potential field contains periods of quasi-stability for brief windows of time (80–120ms) in which the strength of the electric field may vary but the field configuration remains stable (Wackermann et al., 1993). Since the configuration of the electric field at the scalp is independent of the choice of reference electrode, it can be assumed that when a change in topographic configuration arises, it reflects an underlying change in the neural source generators driving the topography, and therefore information processing (Lehmann, 1987). Hence, these periods are known as functional microstates and correspond to epochs of synchronised large-scale neural activity (Lehmann et al., 1987). The analysis of functional microstates has many advantages over the ERP method. For example, ERP analysis of the SPN difference wave may highlight a prolonged amplitude difference between conditions, yet this analysis cannot determine if the differences observed are due to fluctuations in field strength or represent an underlying shift in neural source generators driving the effect (for details see Murray et al. (2009)).

Firstly, paired topographic ANOVA (TANOVA) comparisons (non-parametric randomisation tests) for differences between conditions were performed to determine significant periods of global dissimilarity (DISS). DISS is an index of configuration divergence between two electric fields over time, independent of their strength (Lehmann and Skrandies, 1980). This analysis provides an objective measure of stable topographic differences (we only considered periods of stability more than 30 ms in duration with a p-value below 0.05 for each time point), for example, TANOVA differences over the SPN time window could highlight fundamentally different scalp topographies for noise vs. symmetry conditions. However, it is important to note that TANOVA can only identify when in time topographic differences arise, but does not describe the distribution of these differences. We therefore ran a segmentation analysis (Pascual-Marqui et al., 1995) to identify functional microstates across the ERP epoch. The segmentation procedure involves a Hierarchical Clustering technique (Topographical Atomize and Agglomerate Hierarchical Clustering) performed over grand averaged ERP waveforms (Brunet et al., 2011, Murray et al., 2008). The topographic map at each time point is initially considered a single cluster; the number of clusters/maps is then iteratively reduced into a single unique cluster, which explains the greatest variance in the data over a specific time period – and this is termed a microstate. The optimal number of microstates was determined using the clusters with the minimal cross validation (CV) and maximal Krzanowski-Lai (KL) (Pascual-Marqui et al., 1995, Pegna et al., 2004, Pegna et al., 1997). Cross validation criterion is the ratio between global explained variance and the degrees of freedom for a given set of maps whilst the Krzanowski-Lai criterion computes a quality measure, termed the dispersion (W) curve. Dispersion is then analysed to identify the point where adding more clusters does not increase global quality.

We assessed the statistical validity of our segmentation by determining the amount of variance explained by each microstate in the ERPs of individual participants for all conditions in the time windows used for analysis of the SPN component. Repeated-measures ANOVAs were carried out on the global explained variance to compare the statistical probability of each map explaining each condition. This procedure was completed for each of the three experiments.

We further present images of our microstate segmentation alongside measures of electric field strength (Global Field Power; GFP) to help interpret the interplay between changes in neural source generators of microstates and changes in electric field strength. Global Field Power, the spatial standard deviation of all electrodes at a given time, is a reference independent measure of the strength of electric field differences across the scalp (Skrandies, 1990), and modulations in GFP can occur between conditions without there being any differences in microstates. Similarly, microstate differences between conditions can present with no differences in GFP. Typically, topography remains stable around peaks of GFP. A reduction in GFP without a corresponding modulation in topography can be interpreted as a reduction in the number of synchronously active neural generators (Murray et al., 2008).

## Results

### Experiment 1: Effect of luminance-polarity distribution on the ERP responses to 100% position-symmetric patterns

Fig. 3A shows that performance (% correct answers) increases gradually from anti-symmetric (90.8%), to polarity-grouped (94.8%) and to unsegregated (96.5%) conditions. The data were submitted to a one-way repeated-measures ANOVA. The p-values associated with this and subsequent analyses were those associated with the Greenhouse-Geisser correction for violations of sphericity. For clarity, the original degrees of freedom are reported. The analysis revealed significant differences between the three conditions (F(2, 46) = 17.71, p = 0.001, *η*^2^ = 0.435). Bonferroni-corrected multiple comparison tests showed significant differences between all pair-wise comparisons (anti-symmetric vs. polarity-grouped: t(23) = 4.068, p = 0.001, d = −0.762; anti-symmetric vs. unsegregated: t(23) = 4.459, p = 0.001, d = −1.092 and polarity-grouped vs. unsegregated: t(23) = 3.06, p = 0.01, d = 0.517), confirming that performance increased from anti-symmetric through to unsegregated conditions.

#### ERPs

Fig. 3B shows the grand average ERPs for the position-symmetric (anti-symmetric, polarity-grouped anti-symmetric and unsegregated) and quasi-random (noise and polarity-grouped noise) patterns. The corresponding SPN difference waves (i.e. symmetry – noise) are shown in Fig. 3C. Panels D–F display the topographic differences (symmetry – noise) for anti-symmetric, polarity-grouped and unsegregated conditions between 200 and 600 ms after stimulus onset. Blue areas in Fig. 3D–F indicate that over this time window, the response to symmetry was lower in amplitude than the response to noise over posterior brain regions, with these regions encompassing the areas that electrodes PO7 and PO8 were positioned over.

To examine if grouping by luminance-polarity affects the ERP response to anti-symmetric patterns between 200 and 600ms, we used a two-way repeated-measures ANOVA with factors stimulus type (anti-symmetry, noise) and luminance-polarity grouping (no polarity grouped, polarity-grouped). The analysis revealed a significant main effect of stimulus type (F(1, 23) = 11.5, p = 0.002, *η*^2^ = 0.333), with anti-symmetric conditions producing overall lower ERP amplitudes than noise conditions. There was no significant main effect of luminance-polarity grouping on ERPs and no interaction effect. A separate paired-samples *t*-test between anti-symmetric and unsegregated patterns showed no significant difference in ERPs (t(23) = 0.410, p = 0.685, d = 0.025).

To determine whether the SPN's were significantly different from zero, we conducted one sample t-tests that revealed significant SPNs for all three conditions (anti-symmetric: t(23) = −3.424, p = 0.002, d = −0.698; polarity-grouped anti-symmetric: t(23) = −3.007, p = 0.006, d = −0.613; unsegregated: t(23) = −2.949, p = 0.007, d = −0.601). We then tested for differences in the magnitude of the SPN between these conditions using a one-way ANOVA with factor stimulus type (anti-symmetric, polarity-grouped anti-symmetric, unsegregated). This analysis revealed no significant differences in SPNs for anti-symmetric, polarity-grouped anti-symmetric and unsegregated conditions (F(2,46) = 0.098, p = 0.904). None of the Bonferroni-corrected post-hoc comparisons were significant (p > 0.05).

#### Topographic and microstate segmentation analysis

**Fig. 4:**
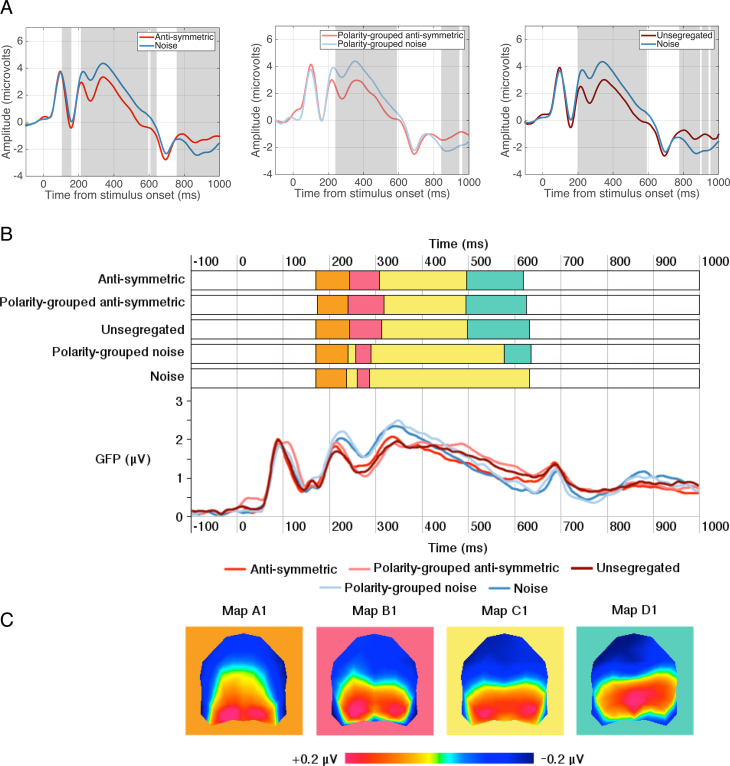
**TANOVA and microstate segmentation analysis for Experiment 1.** (A) Grand average ERPs at electrode sites PO7 & PO8 with light grey areas showing periods of stable topographic differences determined by the TANOVA. (B) Onset and offsets of the stable topographic microstates between 200 and 600ms and Global Field Power waveforms for each condition. Global Field Power waveforms, a measure of differences in the scalp electric field strength, are displayed for a visual comparison to topographic microstates which are independent of field strength. The higher GFP the more stable EEG topography and the higher global excitation. Each map is represented by a different colour. (C) Topographic microstates derived from the segmentation procedure for four maps which best fit the individual subject data between 200 and 600 ms. Topographic maps show the head from above with nasion plotted upward.

To examine differences in topographic maps between symmetry and noise conditions we conducted paired TANOVA comparisons between each luminance-polarity configuration. These analyses revealed significant windows of topographic dissimilarity (anti-symmetric vs. noise, from 213 to 595 ms; polarity-grouped anti-symmetric vs. polarity-grouped noise, 240–590 ms; and unsegregated vs. noise, 200–592 ms) all of which incorporated the time window for analysis of the SPN (200–600 ms – see light gray areas in Fig. 4A). Note that periods of TANOVA differences for each paired comparison occurring after 600 ms incorporate differences occurring after the stimulus offset potential, visible as the negative deflection between 600 and 800 ms. To identify whether periods of topographic divergence assessed by TANOVA displayed different microstates we ran a segmentation analysis (Fig. 4B) which identified four topographic maps between 200 and 600 ms, the time window for analysis of the SPN (Fig. 4C). For all position-symmetric patterns these topographic maps were present in the same order and had similar onset and offsets. However, the durations of the maps in the symmetric pattern conditions differed from those obtained with the noise patterns, with map B1 and D1 of longer durations for the symmetrical than for the noise patterns. Interestingly, map D1 was also present in the polarity-grouped noise which contained a medial axis that separated the two polarities (Fig. 2B) but not for the randomly distributed black and white dots.

The fitting of these maps to individual participants was subjected to a three-way repeated-measures ANOVA with factors map (A1, B1, C1, D1), stimulus type (symmetry, noise) and luminance-polarity grouping (anti-symmetry, polarity-grouped anti-symmetry), analysing the amount of variance each map could explain in individual data. The analysis showed a significant main effect of map (F(3,69) = 6.468, p = 0.007, *η*^2^ = 0.219), with map B1 explaining the greatest proportion of variance across all conditions. The effect of luminance polarity grouping was not significant and there was no interaction between stimulus type and luminance polarity grouping. Critically, we found a marginal interaction between stimulus type and map (F(3,69) = 3.505, p = 0.054, *η*^2^ = 0.132) suggesting that maps B1 and C1 explained more variance for the noise than anti-symmetric patterns, whilst map D1 explained more variance for anti-symmetric than noise patterns. Variance was then examined with a two-way repeated measures ANOVA (map [A1, B1, C1, D1] x stimulus condition [anti-symmetric, unsegregated]) which showed a main effect of map (F(3,69) = 4.038, p = 0.021, *η*^2^ = 0.149) but no effect of stimulus condition or an interaction between them.

### Experiment 2: Effects of luminance-polarity distribution on the ERP responses to 50% position-symmetric patterns

**Fig. 5:**
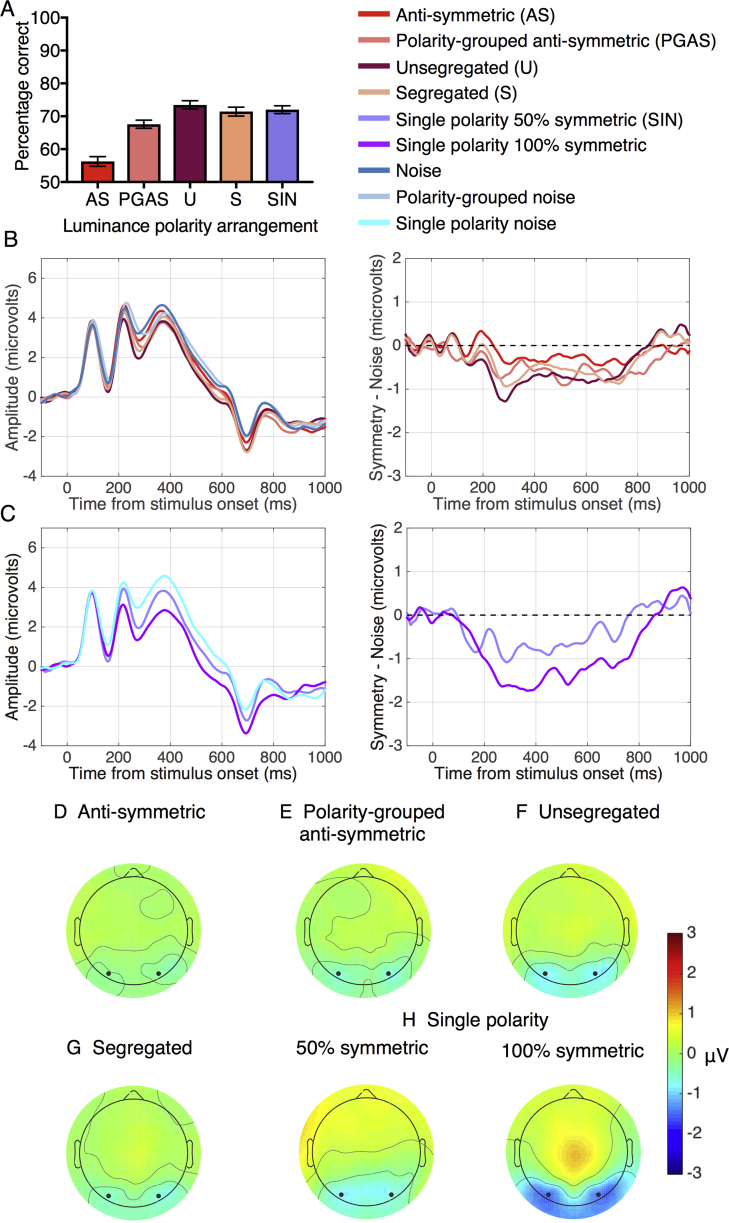
**Results for Experiment 2.** (A) Performance (% correct answers) in the symmetry detection task with segregated, unsegregated, anti-symmetric, polarity-grouped anti-symmetric and single polarity patterns containing 50% position symmetry. (B) Grand-average ERPs (left) and SPN (right) difference wave for anti-symmetric, polarity-grouped anti-symmetric, segregated and unsegregated patterns; (C) Grand-average ERPs (left) and the SPN difference wave for single-polarity patterns containing 50% and 100% position symmetry. Waveforms depict the average of electrodes PO7 and PO8. (D–H) Topographic difference map for anti-symmetric (D), polarity-grouped anti-symmetric (E), unsegregated (F), segregated (G) and single polarity patterns containing 50% and 100% position symmetry (H). Each topographic difference map shows the difference between symmetry and noise in the 200–600 ms time window. Black dots indicate the position of electrodes PO7 and PO8.

Fig. 5A shows performance (% correct answers) for anti-symmetric, polarity-grouped anti-symmetric, unsegregated and single polarity conditions for the 50% symmetric patterns. Performance was highest with unsegregated (73.5%) and gradually declined from single polarity patterns (72.1%), to segregated (71.4%), to polarity-grouped anti-symmetric (67%), reaching chance level with anti-symmetric (56.2%) patterns. A repeated-measures one-way ANOVA showed significant differences between these conditions (F(4,92) = 39.292, p = 0.001, *η*^2^ = 0.631). Bonferroni-corrected multiple comparisons tests showed a number of significant differences between conditions but not between segregated and unsegregated conditions (t(23) = 1.664, p > 0.9, d = 0.316), suggesting that symmetry mechanisms are *not selective* to luminance polarity. Specifically, we found significant differences between anti-symmetric and unsegregated conditions (t(23) = 8.904, p = 0.001, d = −2.523) suggesting that symmetry mechanisms are *sensitive* to luminance polarity (see also Gheorghiu et al., 2016 for a discussion on sensitivity vs. selectivity to luminance polarity and colour), and between anti-symmetric and polarity-grouped anti-symmetric conditions (t(23) = 7.533, p = 0.001, d = −1.701) suggesting that grouping by luminance polarity improves symmetry detection.

#### ERPs

ERPs for Experiment 2 displayed a typical P1-N1-P2 complex for all stimulus conditions. Fig. 5B shows the ERP responses (left panel) and SPN responses (right panel) obtained with anti-symmetric (red), polarity-grouped anti-symmetric (pink), segregated (orange) and unsegregated (dark red) conditions. Segregated and unsegregated conditions produced a comparable SPN suggesting than ERPs are not selective to luminance polarity of the symmetric pattern. Unsegregated patterns produced larger ERPs than anti-symmetric and polarity-grouped anti-symmetric patterns suggesting that ERPs are sensitive to luminance-polarity correlations across the symmetry axis. Fig. 5C shows the ERPs (left panels) and SPN (right panels) responses obtained with single-polarity patterns containing either 50% or 100% position symmetry. A strong SPN was produced for both 50% and 100% position symmetry with the difference in SPN amplitude being larger for 100% than 50% symmetry suggesting that the SPN is modulated by the amount of position symmetry in the stimuli. Topographic plots showed that these ERPs were generated from posterior visual areas (Fig. 5D–H).

To examine the effects of grouping by luminance polarity in anti-symmetric patterns between 200 and 600ms after stimulus, a two-way repeated-measures ANOVA with factors stimulus type (anti-symmetry, noise) and luminance-polarity grouping (no polarity grouping, polarity-grouped) was carried out on the ERP data. The analysis revealed a significant main effect of stimulus type (F(1, 23) = 9.635, p = 0.005, *η*^2^ = 0.295), indicating that anti-symmetric conditions produced on average lower amplitude ERPs than noise conditions. There was no significant main effect of luminance-polarity grouping suggesting that irrespective of whether the pattern has position-symmetry or not, grouping by luminance polarity does not modulate ERPs, and no interaction effect (p > 0.05).

To examine the effect of luminance-polarity distribution across the symmetry axis between 200 and 600ms, a one-way repeated-measures ANOVA was carried out on the ERPs obtained with different luminance-polarity arrangements in position symmetric patterns (anti-symmetric, segregated, unsegregated). The analysis revealed a significant main effect of luminance-polarity arrangement in symmetric patterns (F(2,46) = 3.879, p = 0.033, *η*^2^ = 0.144). Bonferroni corrected multiple comparisons found significant differences between anti-symmetric and unsegregated (t(23) = 3.23, p = 0.011, d = 0.142). No significant differences were found between the other conditions.

Further, we confirmed that the SPNs generated were significantly different from zero using one-sample t-tests conducted for each condition (anti-symmetric: (t(23) = −2.355, p = 0.027, d = −0.480; polarity grouped anti-symmetric: t(23) = −2.387, p = 0.026, d = −0.487; unsegregated: t(23) = −4.390, p = 0.001, d = −0.896; segregated t(23) = −3.220, p = 0.004, d = −0.657). We also tested for differences in the magnitude of SPNs between conditions using a one-way ANOVA with factor stimulus type (anti-symmetric, segregated, unsegregated). This showed that there was a main effect of stimulus type (F(2,46) = 3.879, p = 0.033, *η*^2^ = 0.144). Bonferroni-corrected multiple comparisons showed a significant difference between anti-symmetric and unsegregated conditions (t(23) = 3.23, p = 0.022, d = 0.781) indicating that luminance polarity distribution across the symmetry axis affects the SPN response to position-symmetry.

We also ran a one-way repeated-measure ANOVA to examine differences in ERPs for the single polarity patterns containing 100%, 50% and 0% (noise) position symmetry. The analysis revealed a significant effect of amount of position symmetry (100%, 50%, 0% or noise) (F(2,46) = 25.001, p < 0.001, *η*^2^ = 0.521), with 100% position symmetry eliciting lower ERP amplitude then 50% symmetry and compared to noise.

#### Topographic and microstate segmentation analysis

**Fig. 6:**
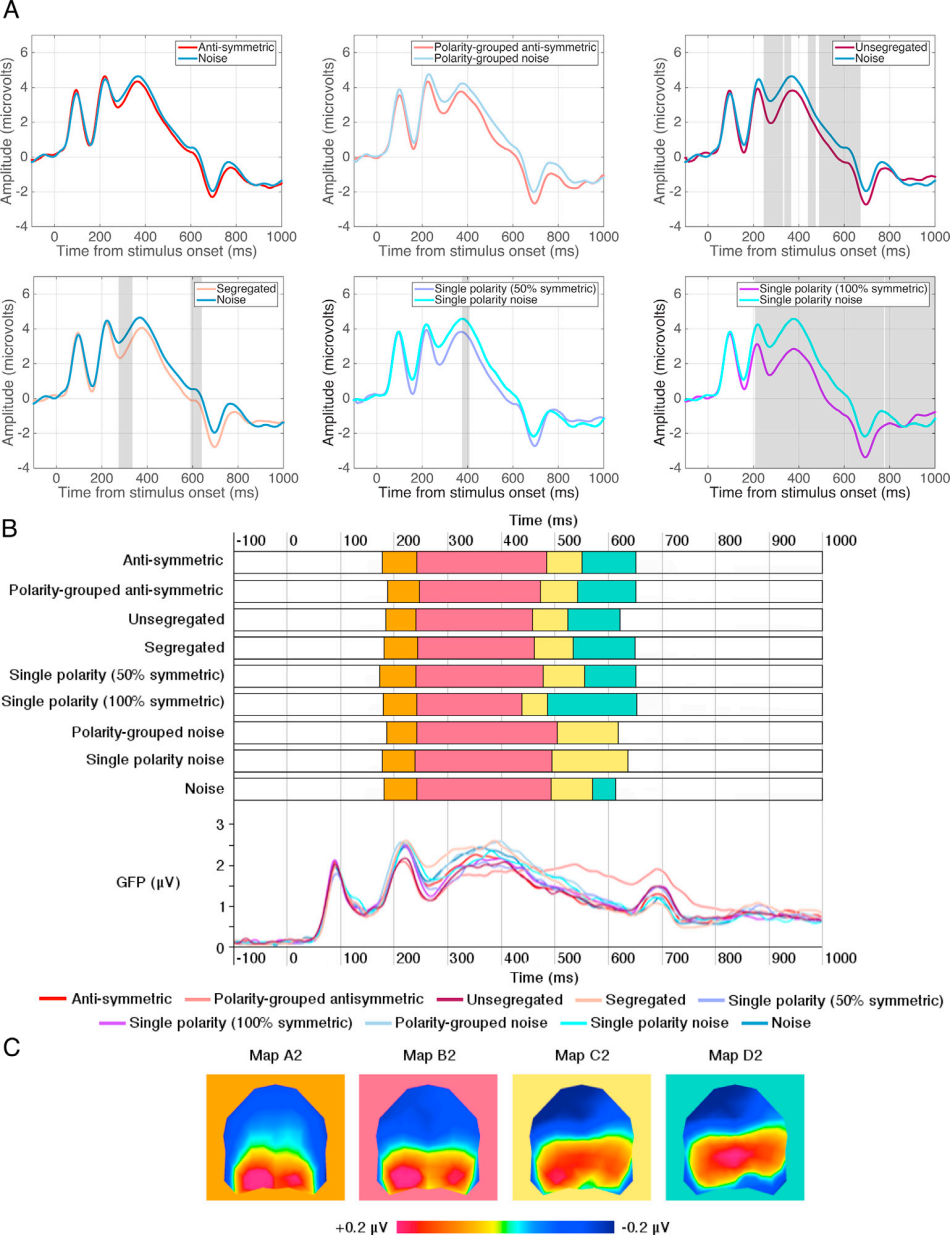
**Topographic and microstate segmentation analysis for Experiment 2.** (A) Grand average ERPs represent the average of posterior electrodes PO7 & PO8, with light grey areas showing periods of stable topographic differences determined by TANOVA. (B) Onset and offsets of topographic microstates between 200 and 600 ms and Global Field Power waveforms for each condition. Global Field Power waveforms, a measure of differences in the scalp electric field strength, are displayed for a visual comparison to topographic microstates which are independent of field strength. The higher GFP the more stable EEG topography and the higher global excitation. Each map is represented by a different colour. (C) Topographic microstates derived from the segmentation procedure for four maps which best fit the individual subject data between 200 and 600 ms. Topographic maps show the head from above with nasion plotted upward.

As in Experiment 1, we conducted paired-samples TANOVA analyses between each symmetric (i.e. segregated, unsegregated, anti-symmetric, polarity-grouped anti-symmetric, single polarity with 50% and 100% position symmetry) and corresponding noise stimuli (Fig. 6A). For the anti-symmetric and noise stimuli, there were no significant periods when topography differed suggesting that the same neural generators produced these ERPs. This was also the case for the polarity-grouped anti-symmetric and polarity-grouped noise. Further, the scalp topography for unsegregated conditions was different from noise in two specific time periods (245–366 ms and 440–672 ms after stimulus onset). In the segregated condition, there was a single time period (275–336ms) displaying topographic differences compared to noise. For the single polarity with 50% position symmetry, a brief period of topographic dissimilarity was found between 352 ms and 384 ms. In comparison, single polarity patterns with 100% position symmetry displayed two periods (207–774 ms and 778–1000 ms after stimulus onset) of significant differences between topographic maps. These results suggest that early differences between noise and 50% position symmetry conditions are observed only for segregated and unsegregated symmetry stimuli, which reflect the trends in behavioural performance in Experiment 2.

Microstate segmentation analysis identified four stable topographic maps all of which showed positivity in posterior scalp locations (Fig. 6C). These four maps occurred in the same order and had similar durations for all conditions except for polarity-grouped noise and single-polarity noise, which were missing Map D2. We assessed the fit of each map and the amount of variance each map explains for individual participant data, with a three-way repeated-measures ANOVA with factors stimulus type (anti-symmetry, noise) x luminance-polarity grouping (no polarity grouping, polarity-grouped) x map (A2, B2, C2, D2). The analysis revealed a significant main effect of stimulus type (F(1,23) = 8.845, p = 0.007, *η*^2^ = 0.278) showing that noise explained more variance than anti-symmetry. There was also a significant main effect of map (F(3,69) = 9.687, p = 0.001, *η*^2^ = 0.296) showing that Map B2 accounted for the most variance in individual participants during the 200–600 ms time window. Further, an interaction between stimulus type x map (F(3,69) = 4.969, p = 0.013, *η*^2^ = 0.178) showed that Map B2 better fits the data for noise compared to anti-symmetry conditions, whilst Maps A2 and D2 explained more of the variance for anti-symmetry than noise. Variance was then examined using a two-way repeated measures ANOVA with factors map (A2, B2, C2, D2) and symmetry condition (anti-symmetric, polarity-grouped anti-symmetric, segregated, unsegregated, single polarity with 50% position symmetry) which found that only the main effect of map was significant (F(3,69) = 11.269, p = 0.001, *η*^2^ = 0.329).

### Experiment 3: Effect of number of colours on the ERP response to symmetry

**Fig. 7:**
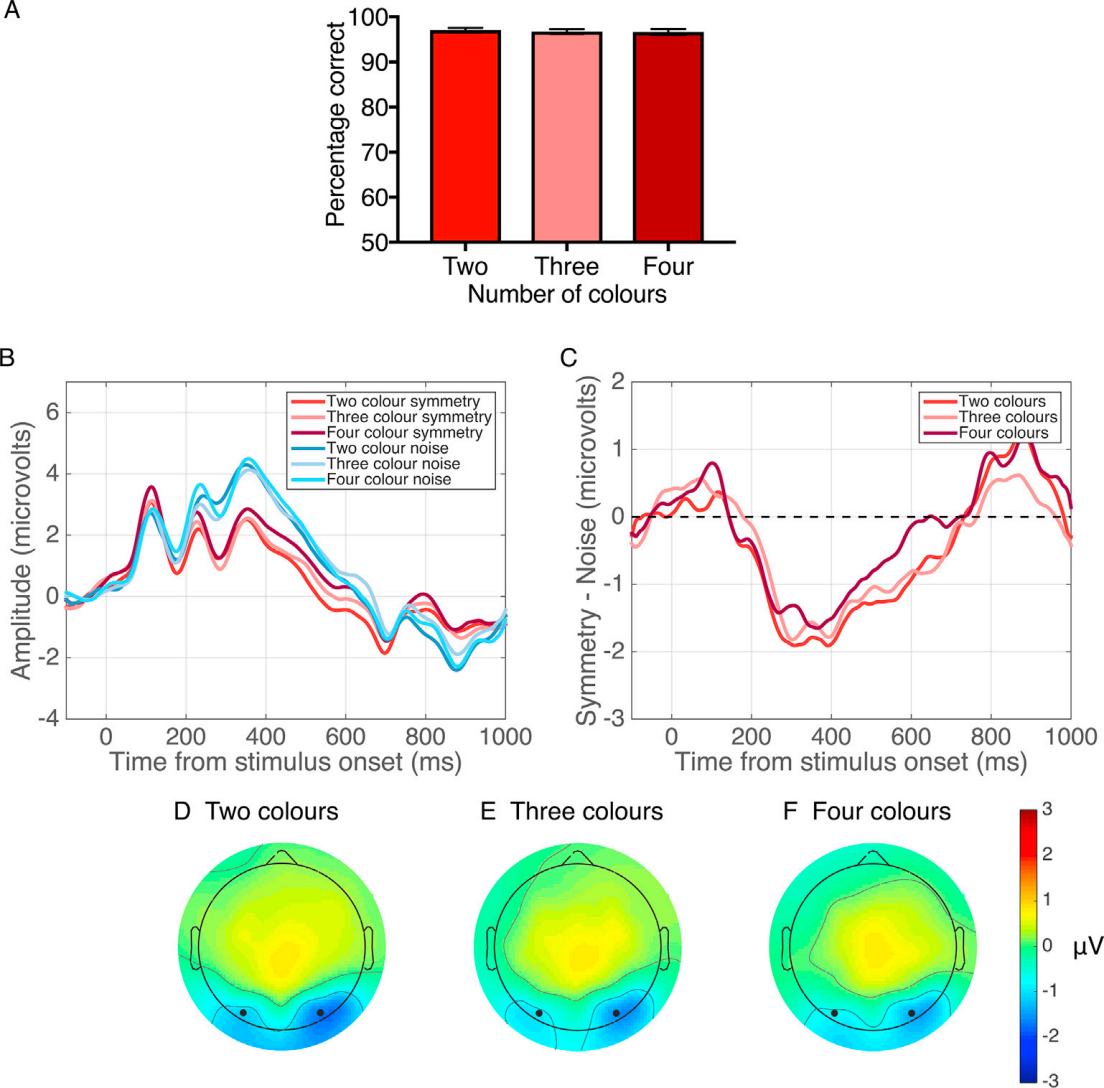
**Results for Experiment 3.** (A) Performance (% correct answers) in the symmetry detection task with two, three and four colours. (B) Grand-average ERPs for two colour symmetry (red) and noise (blue), three colour symmetry (pink) and noise (light blue) and, four colour symmetry (dark red) and noise (cyan) conditions. (C) SPN difference waves (Symmetry–Noise) for each condition. Waveforms depict the average of electrodes PO7 and PO8. (D–F) Topographic difference map for two (D), three (E) and four (F) colours. Each topographic difference map shows the difference between symmetry and noise in the 200–600 ms time window. Black dots indicate the position of electrodes PO7 and PO8.

Fig. 7A shows performance (% correct answers) in the symmetry detection task with perfectly symmetric patterns containing two, three and four colours. A one-way repeated-measures ANOVA indicated similar performance with two (97%), three (96.7%), and four (96.6%) colours (F(2,46) = 0.4985, p = 0.54).

#### ERPs

ERPs and corresponding SPN difference waves are shown in Fig. 7B and C, respectively, for each number of colours condition. The results indicate a slightly reduced SPN for four-colour stimuli, while two and three colour stimuli produced larger SPNs but of comparable magnitude.

A two-way repeated-measures ANOVA with factors of stimulus type (symmetry, noise) and number of colours (two, three, four) revealed a significant main effect of stimulus type with lower ERP amplitudes for position symmetric stimuli over noise (F(1,23) = 15.135, p = 0.001, *η*^2^ = 0.397), and a significant effect of number of colours on ERP mean amplitude (F(2,46) = 3.675, p = 0.032, *η*^2^ = 0.138) with two and three colours producing lower ERP amplitudes than four colours between 200 and 600ms. There was no significant interaction between stimulus type and number of colours (p > 0.05).

One sample t-tests were conducted to examine whether the SPNs were significantly different from zero. These showed that all number of colour conditions produced significant SPNs (two colours: t(23) = −4.677, p = 0.001, d = −0.954; three colours: t(23) = −3.447, p = 0.002, d = −0.621; four colours: t(23) = −2.604, p = 0.016, d = −0.531). However, a one-way ANOVA analysis of the SPN responses indicated no significant differences between the magnitudes of the SPNs obtained with two, three and four colours (F(2,46) = 0.889, p = 0.413, *η*^2^ = 0.008). None of the Bonferroni-corrected post-hoc comparisons were significant (p > 0.05). Fig. 7D–F show topographic difference plots for each number of colours conditions corresponding to 200–600 ms after stimulus onset. Blue areas indicate that the response to symmetric patterns was lower in amplitude than with noise patterns and, for all three conditions, the SPN was produced in posterior brain regions.

#### Topographic and microstate segmentation analysis

**Fig. 8:**
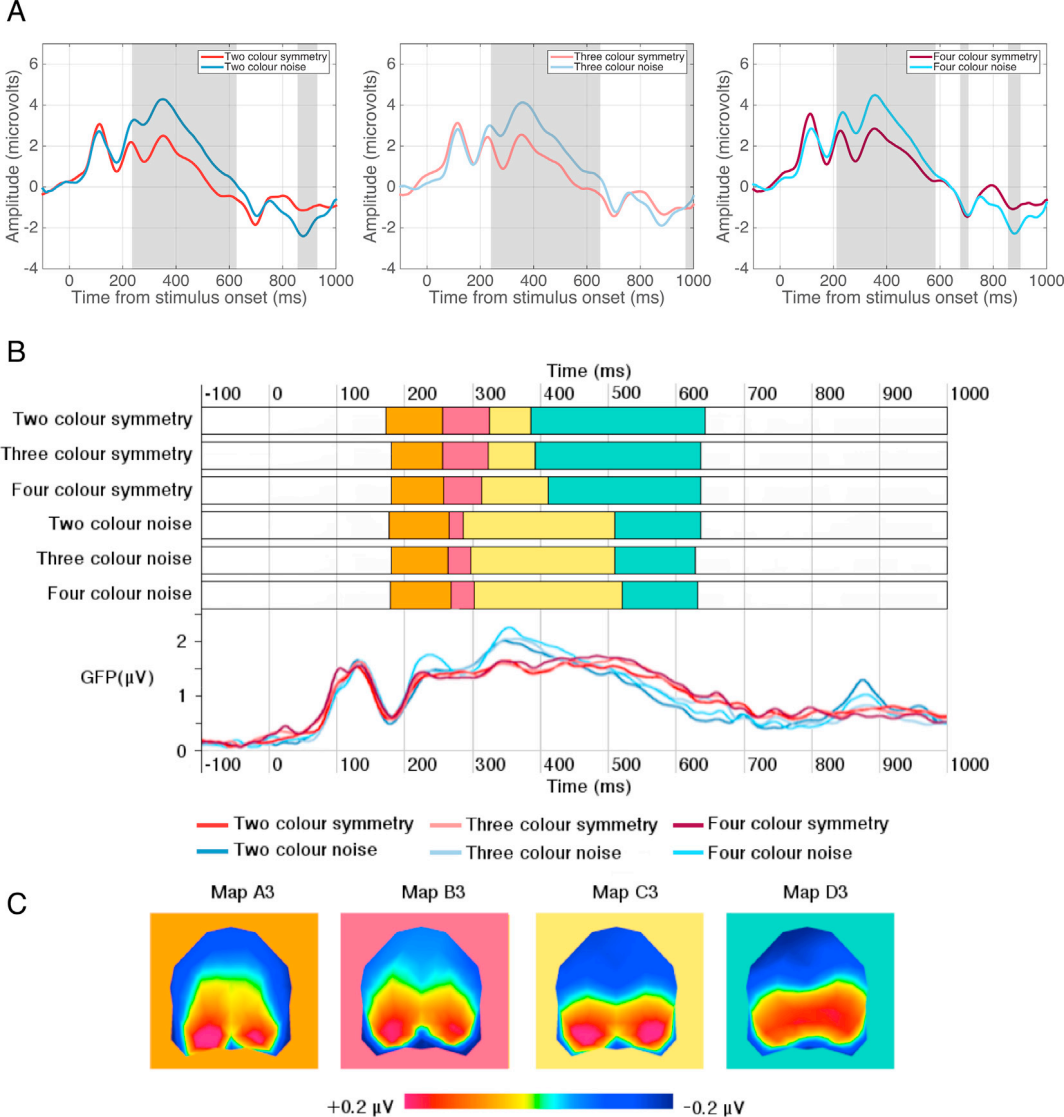
**TANOVA and microstate segmentation analysis for Experiment 3.** (A) Grand average ERPs represent the average of posterior electrodes PO7 & PO8, with light grey areas showing periods of stable topographic differences determined by TANOVA. (B) Onset and offsets of the stable topographic microstates between 200 and 600 ms and Global Field Power waveforms for each condition. Global Field Power waveforms, a measure of differences in the scalp electric field strength, are displayed for a visual comparison to topographic microstates which are independent of field strength. The higher GFP the more stable EEG topography and the higher global excitation. Each map is represented by a different colour. (C) Topographic microstates derived from the segmentation procedure for four maps which best fit the individual subject data between 200 and 600 ms. Topographic maps show the head from above with nasion plotted upward.

A paired TANOVA comparison highlighted a significant window of topographic dissimilarity between 200 and 600 ms after stimulus onset between symmetric and noise patterns in each of the two (236–628 ms), three (241–651 ms), and four (212–583 ms) number of colours conditions (Fig. 8A). These periods of dissimilarity correspond to the time window of analysis of the SPN.

Microstate segmentation analysis identified four distinct maps (A3, B3, C3, and D3) between 200 and 600 ms for each number of colour conditions (Fig. 8B). These maps were present in the same order in all conditions but there were differences in the maps' duration between the symmetry and noise conditions. For the symmetrical patterns, map B3 and D3 had a longer duration whilst map C3 had a shorter duration compared with the noise patterns. We compared the fit of each map to individual participant data in the 200–600 ms time window using a stimulus type (symmetry, noise) x number of colours (2, 3, 4) x map (A3, B3, C3, D3) repeated-measures ANOVA performed on the variance explained by each map. The analysis found a significant main effect of Map (F(3,69) = 14.174, p = 0.001, *η*^2^ = 0.381) showing that Map C3 explained the greatest amount of variance across all conditions. There was a significant stimulus type × map interaction (F(3,69) = 8.175, p = 0.004, *η*^2^ = 0.262) suggesting that Maps B3 and D3 better explain the symmetry than the noise conditions whilst Maps A3 and C3 better explain the noise than the symmetry conditions.

## Discussion

We examined across three experiments how luminance-polarity distribution across the symmetry axis, grouping by luminance-polarity in anti-symmetric and symmetric patterns, and the number of colours in the stimuli affect symmetry detection and the SPN response to symmetry. We found: (**A**) superior performance for luminance-polarity-grouped compared to classic anti-symmetric patterns suggesting that symmetry perception in anti-symmetric patterns can benefit from grouping by luminance polarity in both 50% and 100% position-symmetric conditions. (**B**) superior performance for the segregated and unsegregated condition in comparison to both types of anti-symmetric patterns confirming previous findings that symmetry is sensitive to luminance polarity correlations across the symmetry axis for both 50% and 100% position-symmetric patterns. (**C**) In contrast to behavioural performance, the SPNs obtained with 100% position-symmetric unsegregated, anti-symmetric and polarity-grouped anti-symmetric patterns were comparable in amplitude. Critically, for perfect position-symmetry conditions compared to noise, we show topographic differences similar in duration to the observed SPN component. These differences are explained by the late presence of a functional microstate in the SPN time window that appears in symmetric conditions (map D1) and polarity-grouped noise. (**D**) For patterns with 50% position symmetry, SPN amplitude was reduced for anti-symmetric compared to unsegregated stimuli, which reflected behavioural performance on the task. Topographic differences between 50% position symmetry and noise conditions were only observed for segregated and unsegregated conditions, however the presence of a microstate for all symmetry conditions (map D2), similar to Experiment 1, was again observed. (**E**) The comparable SPN amplitude obtained with segregated and unsegregated conditions in patterns with 50% position symmetry reflect similar behavioural performance obtained in these conditions, thus confirming previous findings that symmetry mechanisms are not gated by or selective to luminance-polarity. (**F**) As for chromatic patterns containing two, three and four-colours, we found a significant effect of number of colours on the ERP responses but not on the SPN amplitude. The SPN amplitudes were comparable regardless of the number of colours, and this was also reflected by similar behavioural performance. Again, topographical analysis confirmed that the SPN difference wave reflects different underlying neural source generators for symmetry compared to noise conditions, and the presence of map D3 for symmetry was longer in duration compared to Experiments 1 and 2.

Across all three experiments we demonstrate that the SPN difference wave is sensitive to the amount of symmetry in the stimulus (100% vs. 50% position symmetry) and to how luminance polarity is distributed across the symmetry axis, and that the sustained nature of the SPN difference may coincide with the late onset of a topographic microstate sensitive to symmetry axis and a mid-line axis defined by luminance-polarity grouping.

Our behavioural results complement previous findings exploring the role of luminance polarity in symmetry detection showing that observers experienced great difficulty perceiving symmetry in the anti-symmetric patterns (Brooks and van der Zwan, 2002, Zhang and Gerbino, 1992). These results suggest that symmetry mechanisms are sensitive to luminance-polarity, confirming previous findings by Troscianko (1987) and Gheorghiu et al. (2016). We also found that performance improved significantly with luminance-polarity-grouped anti-symmetric patterns suggesting that grouping by luminance polarity facilitates symmetry detection in anti-symmetric stimuli. This finding goes against previous reports that grouping by luminance polarity does not improve performance (Zhang and Gerbino, 1992).

*Why did we find no differences in the magnitude of the SPN in Experiment 1?* When symmetric patterns contain 100% position symmetry, ERP and microstate responses to symmetry were not sensitive to luminance polarity correlations across the symmetry axis. This result complements Makin et al. (2016), who found that one-fold symmetry, equivalent to our unsegregated condition, and anti-symmetric patterns produced a comparable SPN response. However, 100% position-symmetric patterns may elicit an optimal symmetry detection response, possibly involving high-level extra-striate brain areas (Sasaki et al., 2005, Tyler et al., 2005), irrespective of luminance polarity. In addition, fully symmetric unsegregated patterns do not allow for symmetric and noise dots to be separated (i.e. segregated or grouped) by luminance polarity. Therefore, in contrast to Makin et al. (2016), Experiment 2 utilised a segregated condition in which symmetry and noise dots were grouped by luminance polarity, with polarity of the symmetric patterns being randomly drawn from white and black in different trials. Note that the amount of position symmetry in all these conditions (segregated, unsegregated, anti-symmetric, polarity grouped anti-symmetric and single polarity) was always the same (50% position symmetry).

In Experiment 2, we demonstrated that single polarity patterns with 50% position symmetry produce a robust, although slightly reduced SPN response than 100% position symmetry patterns, a finding which compliments Palumbo et al. (2015). Further, Palumbo et al. found a graded increase in the amplitude of the SPN in line with the increase in the amount of position symmetry present in the stimulus. It may be the case that differences in the amplitude of the SPN for stimuli containing 50 and 100% position symmetry found in the present study could reflect the slightly reduced behavioural performance with 50% position symmetric patterns. More importantly, our results also indicate that both behavioural performance and ERP amplitude obtained with segregated and unsegregated patterns were comparable, thus supporting previous behavioural findings indicating that symmetry mechanisms are *not selective* to luminance polarity (Gheorghiu et al., 2016, Morales and Pashler, 1999) that is, there are no separate on and off luminance-polarity channels for symmetry. This finding, which has not previously been shown with ERPs, suggests that symmetry detection mechanisms pool both luminance-polarities into one channel, and thus, extra-striate visual areas sensitive to symmetry are not gated by luminance polarity.

The existing literature is equivocal as to the role of luminance-polarity in symmetry perception and on the SPN response to symmetry. While Makin et al. (2016) have suggested that the SPN response to symmetry may feed flexibly on both first (luminance sensitive) and second order (contrast sensitive) channels, other psychophysical studies have suggested that equal sensitivity to symmetry and anti-symmetry is the result of similar responses from second-order channels (Saarinen and Levi, 2000, Tyler and Hardage, 1996, Wenderoth, 1996, Zhang and Gerbino, 1992). However, Mancini et al. (2005) showed that the *equal sensitivity to symmetry and anti-symmetry* found by previous studies might not be due to the involvement of second-order channels. Instead, these authors suggest that sensitivity to symmetry arises from spatial-filtering models involving quasi-linear channels whereas sensitivity to anti-symmetry arises from *attentional mechanisms* that operate only in sparse displays. In other words, anti-symmetry is only detected under conditions favourable to selective attention that registers the positional symmetry of individual dots that differ in luminance-polarity. This is also consistent with findings from Morales and Pashler (1999). For our briefly presented stimuli (500 ms), one would not expect this attentional resource to benefit performance with anti-symmetric (and symmetric) patterns. Thus, our findings showing better performance and larger SPNs with unsegregated than anti-symmetric patterns are consistent with Mancini et al. (2005). Our microstate segmentation analysis suggests for the first time that these luminance polarity arrangements engage the same neural mechanisms, but differ in strength according to luminance-polarity distribution across the symmetry axis.

Finally, we measured ERPs in response to multi-colour symmetric patterns (Experiment 3) and found a significant main effect of the number of colours on the ERP amplitude but not on SPN. The SPN difference wave was not modulated by the number of colours in the stimuli, with two, three and four colour patterns all producing a comparable SPN, in agreement with behavioural performance. Further, no microstate was found that was specific to the number of colours in the stimuli. The finding that ERP amplitude was modulated by the number of colours might be explained by the differences in stimulus saliency, with matching being accomplished more easy and rapid when there are fewer colours. For example, Morales and Pashler (1999) found that reaction times were slower and less accurate for four-colours than two-colours patterns, suggesting that symmetry in multi-colour patterns could only be detected by *switching attention* from one colour to the next. Thus, it remains possible that attentional mechanisms for features/colour need longer time (than 500 ms) to be recruited and thus, contribute less to the signal (e.g. change the gain of the colour-sensitive channels) in four-colour stimuli compared to two and three-colour conditions.

*What do our microstate findings mean for our understanding of the neural mechanisms involved in symmetry perception?* Microstate segmentation analysis from our three experiments all produced a number of stable topographic maps between 200 and 600ms. Changes between each stable microstate in sequence have been claimed to reflect changes in information processing (Lehmann et al., 1998). Across all three experiments we observed the late onset of a map sensitive to symmetry in the stimulus, at both 100% and 50% positionsymmetry. Earlier in the time window of the SPN, functional microstates are similar across all three experiments for symmetry and noise conditions (Maps A, B, and C). This suggests that early SPN differences found here are not driven by symmetry sensitive mechanisms per se, but a general form of pattern perception that is enhanced by the form/gestalt of the stimulus (e.g. when symmetry or luminance-polarity grouping occurs). The presence of map D1 and D2 in Experiments 1 and 2 for all position-symmetric patterns (and polarity grouped noise which contains a medial axis defined by luminance-polarity change), suggests that any symmetry sensitive mechanisms onset much later in the ERP, and hence this may explain the prolonged nature of the SPN difference observed in multiple ERP studies (Makin et al., 2014, Makin et al., 2016, Palumbo et al., 2015, Wright et al., 2015). During Experiment 3, microstate D3, was highly similar in shape to D1 and D2: onsets very early (Fig. 8B) and is present in noise conditions also. However, this map was longer in duration for symmetry compared to noise conditions, even if the number of colours did not modulate performance on the task.

If we have identified a symmetry sensitive microstate in all experiments, why is it also present in some noise conditions across the three experiments? One criticism of the microstate segmentation approach is that the hierarchical clustering method allows only one microstate to exist for a specific time point, i.e. the microstate that explains the most variance in the data set. However, this is not to say that other microstates would not explain a significant proportion of the variance in that time period. For example, one microstate could explain 25% of the variance in the sample, and therefore claim a specific time period, since no other microstates would explain enough of the variance in the sample. However, there may have been a second microstate in that time period which explained 20% + of the variance in the sample but by virtue of the clustering method employed, was ultimately removed. This may explain the presence of map D in our polarity-grouped noise condition in Experiment 2 and the noise conditions in Experiment 3. For the time period covered by map D in these conditions, map C may also have explained a large amount of variance, however, this was exceeded by the amount of variance explained by map D.

*To conclude:* We have found superior performance for the polarity-grouped anti-symmetric patterns compared to anti-symmetric conditions, confirming that symmetry detection with anti-symmetric patterns can benefit from grouping by luminance-polarity. The amplitude of ERPs obtained with 50% symmetric unsegregated patterns were lower than those obtained with anti-symmetric patterns which reflects observers' performance in these conditions. Microstate segmentation analysis suggested that luminance polarity arrangements engaged the same processes but to differing degrees. Our results showed that the SPN difference is reduced by the amount of position symmetry and luminance-polarity mismatch across the symmetry axis, but not modulated by the number of colours in the stimuli. Furthermore, our results suggest that the sustained nature of the SPN difference wave may be driven by the late onset of symmetry sensitive mechanisms, evidenced by a topographic microstate always present in cases of position symmetry. These findings emphasise not only the role of position symmetry, but the importance of visual feature matching (e.g. luminance polarity, colour) across the symmetry axis.
